# Data-driven assessment of Apulian road network resilience: Bridge unavailability and inner municipality isolation impact

**DOI:** 10.1371/journal.pone.0333308

**Published:** 2025-10-10

**Authors:** Niloofar Kheirkhahan, Loredana Bellantuono, Nicola Amoroso, Roberto Cilli, Lorenzo De Biase, Valentina Lucaferri, Alfonso Monaco, Chiara Ormando, Ester Pantaleo, Domenico Pomarico, Sabina Tangaro, Alberto Tofani, Roberto Bellotti

**Affiliations:** 1 Dipartimento Interateneo di Fisica “M. Merlin”, Università degli Studi di Bari Aldo Moro, Bari, Italy; 2 Dipartimento di Biomedicina Traslazionale e Neuroscienze (DiBraiN), Università degli Studi di Bari Aldo Moro, Bari, Italy; 3 Istituto Nazionale di Fisica Nucleare (INFN), Sezione di Bari, Università degli Studi di Bari Aldo Moro, Bari, Italy; 4 Dipartimento di Farmacia - Scienze del Farmaco, Università degli Studi di Bari Aldo Moro, Bari, Italy; 5 ENEA, Italian National Agency for New Technologies, Energy and Sustainable Economic Development, Bologna Research Center, Bologna, Italy; 6 ENEA, Italian National Agency for New Technologies, Energy and Sustainable Economic Development, Casaccia Research Center, Rome, Italy; 7 Dipartimento di Scienze del Suolo, della Pianta e degli Alimenti, Università degli Studi di Bari Aldo Moro, Bari, Italy; Fuzhou University, CHINA

## Abstract

Road networks are crucial for the movement of resources, passenger transportation, and supply chains. In seismically active areas like Italy, earthquakes can compromise road infrastructure, leading to structural failures and connectivity disruptions. Bridges, vital for travel and emergency response, are especially vulnerable to these extreme events, making their maintenance and recovery crucial for preserving transport efficiency. This study examines the resilience of the Apulian road network against bridge failures by assessing seismic hazards, the structural vulnerability of each bridge to seismic actions, and the systemic consequences of its disruption. A bridge criticality score is defined to support data-driven decision-making for bridge maintenance and recovery. This novel quantitative metric integrates seismic hazard data at each bridge site, fragility curves, and topological complex network analysis to provide a comprehensive evaluation of bridge criticality. Additionally, the risk of isolation for inner municipalities due to bridge disruptions is assessed using centrality metrics. By combining the bridge criticality score with an emphasis on inner municipalities, this approach offers valuable insights to improve road network resilience, mitigate isolation risks, and promote territorial sustainability in earthquake-prone zones.

## Introduction

Modern societies rely on the efficient operation of critical infrastructures, including transportation networks, power grids, and communication systems, which are essential for economic prosperity, urban connectivity [1], and equitable access to vital services like healthcare, especially for vulnerable individuals [2]. Disruptions to these systems, whether due to natural hazards or technological failures, can trigger cascading effects with severe consequences [3]. Road networks are vital for resource movement, passenger transit, and supply chains, fostering socioeconomic growth through improved market access and business opportunities [4,5]. In Italy, where roads remain the main mode of transportation, ensuring network continuity is crucial [6,7]. However, these networks are highly susceptible to disruptions due to urbanization, natural disasters, and technological failures [8,9], with earthquakes and landslides being among the most significant threats[10]. The resilience of a transport network is defined by its ability to withstand disruptions, maintain functionality, and recover quickly [11,12]. To effectively assess and mitigate vulnerabilities, it is crucial to understand the inherent topological properties of large-scale road networks. For instance, the Global Road Network (GRN), comprising over 14 million km of major roads, exhibits distinct scaling structures with heavy-tailed road length distributions [13]. This scale-free behavior, typical of many transport infrastructures, makes them resilient to random failures but notably vulnerable to targeted attacks [14]. In seismically active regions such as Italy, earthquakes pose a significant threat to road infrastructure, potentially causing structural failures such as deck displacement and foundation instability, leading to severe connectivity disruptions [15]. To assess these risks, Zhou et al. applied percolation theory to examine the impact of earthquakes on network functionality [16], while Padgett et al. linked seismic risk assessments to repair strategies to support post-earthquake recovery planning [17].

Bridges are crucial for connectivity and serve as critical lifelines of transportation networks [18]. Bridges are particularly vulnerable to extreme events [10], which makes their maintenance and recovery essential to preserve or restore transport efficiency [19]. Unlike urban areas with alternative routes, rural and less connected regions depend on key links. Thus, a critical bridge loss may severely limit their accessibility, disrupting essential services and hindering emergency response and rescue actions [20].

Assessing seismic risk and resilience requires analyzing the structural properties of a network and the impact of seismic hazards. Recent studies have incorporated seismic risk indicators into road network models to evaluate the infrastructure’s vulnerability and performance under seismic stress. In particular, centrality metrics have been extensively adopted to pinpoint links whose failure would disproportionately impact network accessibility [21], optimize post-earthquake emergency response logistics [22], and quantify systemic risk by combining network analysis with the seismic vulnerability of specific components like bridges [23]. Remarkably, Ferrario et al. analyzed electrical power networks by integrating Energy Not Supplied indicators and outage duration with topological metrics [24]. Their findings show that although centrality measures provide some predictive insights, they do not fully capture seismic risk, emphasizing the importance of combining network topology with hazard-specific risk assessments. Since the impact of an earthquake depends not only on network connectivity but also on the intensity of ground shaking, the incorporation of seismic hazard parameters is essential to improve resilience evaluations and develop effective mitigation strategies in earthquake-prone regions.

In this research, the resilience of the Apulian road network to bridge failures is evaluated, and a quantitative tool is developed to support data-driven decisions on prioritizing maintenance and repair interventions. The analysis focuses on bridges located in the Foggia province. This area warrants a thorough assessment because, in the first months of 2025, it has been affected by seismic events and, in addition, has a road network that includes numerous bridges of varying ages, materials, construction types, seismic design prescriptions, and road classifications. These bridges serve different types of roads, and their failure can alter connectivity to varying degrees. Moreover, the Foggia province includes several municipalities classified as “inner areas” facing demographic decline and socioeconomic challenges due to their remoteness from essential services. Inner-area municipalities are particularly vulnerable to bridge disruptions due to their dependence on limited, often aging, infrastructure and geographic isolation, exacerbating the impact of service interruptions [25].

In this study, the Apulian road network is embedded into the physical space using Geographic Information Systems (GIS), which allows spatial relationships within the infrastructure to be highlighted [26]. To measure the effect of bridge disruptions on road connectivity, a novel buffer zone methodology is introduced, grounded in Tobler’s First Law of Geography, which states that the impact of nearby features is stronger than that of distant ones [27]. This approach quantifies the “spillover effect” of bridge removal throughout the network as a drop in global efficiency at varying distances from disruption, capturing the cascading effects of bridge failures and their spatial propagation. The proposed framework is further motivated by the outcomes of Kilanitis and Sextos [28], who used percolation-based simulations to show how local bridge failures can trigger widespread accessibility loss, and Sun et al. [29], who found that the severity of efficiency loss depends on network topology and redundancy.

The outcomes of the global efficiency drop analysis are integrated with data on regional seismic hazards and the structural vulnerability of individual bridges. Seismic risk assessment can be performed at varying levels of detail depending on data availability [30]. For preliminary analyses, empirical fragility curves from the literature may be used to estimate post-earthquake damage states [31,32]. With additional information, finite element models can be developed to derive structure-specific fragility curves, accounting for deterioration [33] and soil–structure interaction effects [34]. Where monitoring systems are installed, refined models further enhance reliability [35]. In this study, due to limited data availability, empirical fragility curves are used to assess the damage and derive a functionality level, representing the residual functionality after the structural damage caused by an earthquake [32]. This combined approach allows for a comprehensive evaluation of bridge criticalities by assessing seismic hazards, the structural vulnerability to seismic actions of each asset, and the network-level consequences of their failures. While seismic hazard and structural vulnerability of the bridge directly contribute to the risk of each bridge, the global efficiency drop represents an indirect consequence of bridge failures and quantifies the systemic impact of such events on the road network.

**Fig 1:**
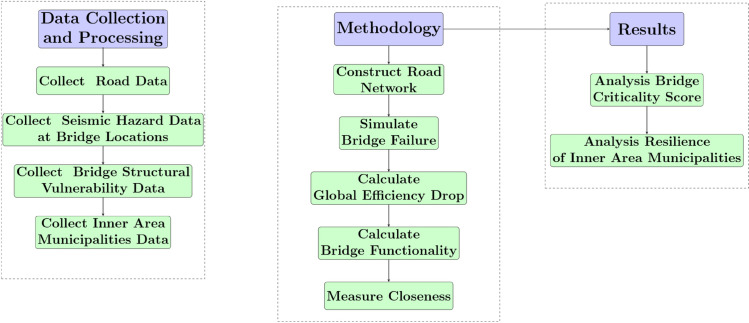
Workflow of the research framework.

The article is structured as follows. In the Results section, a data-driven tool is developed to provide a method to prioritize bridge maintenance and recovery based on the systemic impact of disruptions on network efficiency, bridge seismic hazard, and structural vulnerability. In the same section, the effects of bridge disruptions on the connectivity of inner-area municipalities are also measured. In the Discussion section, implications of the results for resilient planning and strategies against earthquake hazards are explored, and possible directions to improve the proposed framework are highlighted. The Materials and Methods section outlines the approach used to assess the resilience of the Apulian road network in the event of a bridge disruption, quantify bridge criticalities, and examine the vulnerability of inner area municipalities. The research workflow is illustrated in Fig 1.

## Results

This study analyzes the resilience of the Apulian road network in the province of Foggia, with a specific focus on the impact of bridge failures caused by earthquakes. This area, which was affected by seismic shakes at the beginning of 2025, is home to numerous bridges with varying construction materials, seismic designs, span continuity, types of piers, and ages. In addition, Foggia province includes several municipalities in the inner areas and urban centers at risk of depopulation due to limited access to infrastructure and essential services. To quantify the systemic impact of bridge failures, this study uses global efficiency, a graph theory metric that measures the network’s ability to connect nodes through short paths (see Materials and Methods). Specifically, the research focuses on the drop in global efficiency of the surrounding road network following the failure of each bridge. Additionally, the present study introduces a new metric, bridge criticality score, that combines the network impact of bridge failures, quantified by the drop in global efficiency, with the bridge functionality level, determined from fragility curves based on its structural vulnerability and the seismic hazard of its location. Finally, the research evaluates the impact of bridge removals on the connectivity of inner-area municipalities.

### Systemic impact of bridge disruption on network connectivity

**Fig 2:**
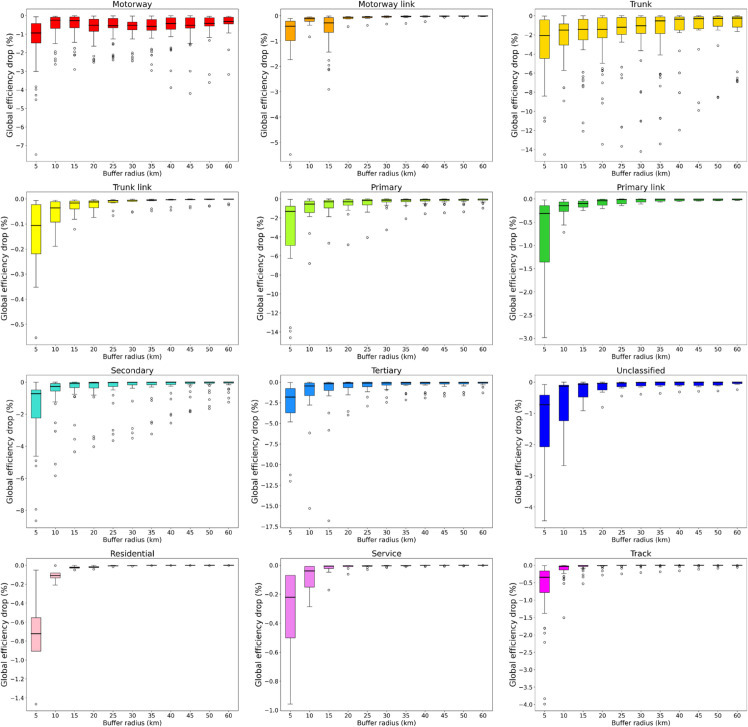
Efficiency drop due to bridge removal. Box plots show the distribution of relative efficiency drop in sub-networks within a radius *R* around the removed bridge, categorized by road type.

**Fig 3:**
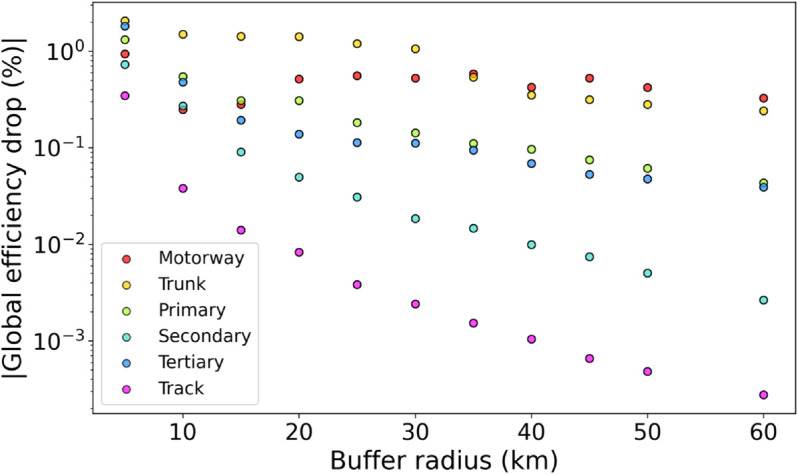
Absolute values of the median relative efficiency drops for specific road types, expressed as a function of the radius around a removed bridge. Only the road types that feature more than 20 bridges are reported.

Applying complex network properties to road network analysis for prioritizing bridge maintenance and repairs is a promising approach [36], but it requires significant computing power due to the complexity of the infrastructure. Specifically, two scenarios must be simulated: one with the network intact and another with the network disrupted by the removal of a bridge. To mitigate the computational burden, this research focuses on a sub-network that includes the nodes within a specified distance from each bridge, along with the links that connect them. Then, the change in global efficiency before and after bridge removal is evaluated. The process repeats by progressively increasing the radius around the bridge in 5-km increments, from 5 to 50 km, with an additional step of 60 km to capture broader network effects. For each bridge and each radius *R*, the relative efficiency drop

$$D(R)=\frac{E_{\text{glob}}(R)-E_{\text{glob}}^{0}(R)}{E_{\text{glob}}^{0}(R)}$$ (1)

is evaluated, where $E_{\text{glob}}^{0}(R)$ and $E_{\text{glob}}(R)$ are the global efficiencies of the selected sub-network (see Materials and Methods for a definition) before and after bridge removal, respectively. This approach reduces computational complexity, enabling a more effective analysis of large-scale road networks [37] while examining how the efficiency drop depends on the distance from the disruption. The graph shown in Fig 2 presents separate boxplots for each bridge type, showing the distributions of relative efficiency drop resulting from bridge removal, evaluated within sub-networks contained in a circle of radius *R* centered on the removed bridge. The results in Fig 3 show how the median relative efficiency drop approaches zero as the distance *R* from a disrupted bridge increases. At short distances, most road types, except for tracks, show similar impacts. However, beyond 40 km, a clear ranking in road resilience becomes evident. Motorways and trunk roads experience the most significant efficiency losses at greater distances, with the magnitude of the effects stabilizing above 0.1%. This reflects their crucial role in long-distance travel: when these roads are disrupted, the effects spread over a wider area. In contrast, disruptions on shorter roads tend to have more localized consequences, with their efficiency losses fading more quickly as distance increases. This suggests that their higher redundancy and more circumscribed territorial coverage contribute to limiting the extent of the damage [38]. In particular, when disrupted, bridges on tracks have the least impact on efficiency at any distance, and the effect decreases with distance at the fastest rate. This reinforces the idea that roads with localized functions and multiple alternative routes are more resilient to disruptions [39].

To better understand the implications of the relative efficiency drop *D*(*R*) for bridges, this metric is compared with a commonly used network indicator: edge betweenness. Edge betweenness quantifies the extent to which a given link (in this case, a bridge) lies on the shortest paths between other pairs of nodes in the network, thus reflecting its importance in connecting different parts of the system. A detailed definition of edge betweenness is provided in the Materials and Methods section. For each of the 369 bridges in Foggia province included in this study, the Pearson correlation between *D*(*R*) and the edge betweenness *b*(*R*) is computed, evaluating metrics on the road sub-network of radius *R* centered on the bridge. This analysis is performed for all *R* values considered in the present study. The results are presented in S1 Table in S1 File.

For almost all *R* values, the relative efficiency drop *D*(*R*) caused by a bridge failure and the corresponding bridge’s betweenness *b*(*R*) show statistically significant negative correlations. This outcome aligns with expectations: bridges with higher betweenness values play a more pivotal role in ensuring network connectivity, and their inaccessibility consequently leads to a more pronounced decline in overall network efficiency. The intensity and statistical significance of this correlation vary weakly with the distance *R* from the bridge, though no strong trend is observed. These consistent negative correlations confirm the relationship between a bridge’s structural importance (as measured by betweenness) and its impact on network resilience following failure.

### Bridge functionality: Combining seismic hazard and structural vulnerability

**Fig 4:**
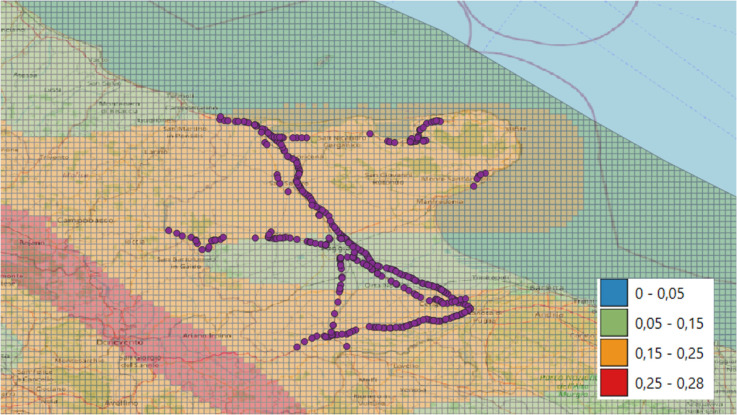
Seismic hazard of the bridges. The figure was created using the open source tool QGIS with OpenStreetMap as the basemap [42]. The seismic hazard values refer to a return period of 475 years, per the Italian Building Code [43].

Bridge functionality represents the ability of a bridge to perform its intended purpose if subject to a seismic event. Functionality is quantified on a continuous scale, reflecting varying levels of performance degradation. Specifically, it is a probabilistic measure that reflects the likelihood that the bridge will maintain a certain level of serviceability given the expected seismic hazard [40] (see Materials and Methods). To evaluate the expected seismic hazard, the seismic zoning of the region is particularly relevant. According to the OPCM 3274/2004 [41], the Italian territory is classified into four seismic zones, with Zone I indicating the highest hazard and Zone IV the lowest. These zones are defined based on Peak Ground Acceleration (PGA) thresholds: Zone I (>0.25g), Zone II (0.15–0.25g), Zone III (0.05–0.15g), and Zone IV (<0.05g). As shown in Fig 4, most considered bridges are located in Zone II, while a smaller portion falls within Zone III.

**Fig 5:**
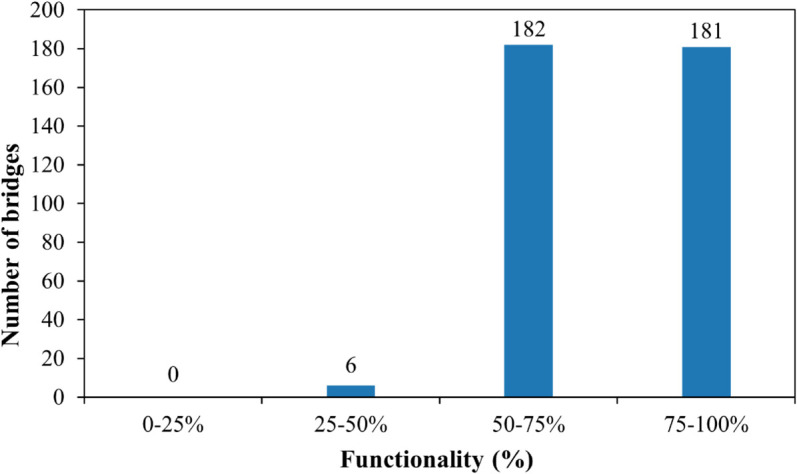
Functionality of the considered bridges.

**Fig 6:**
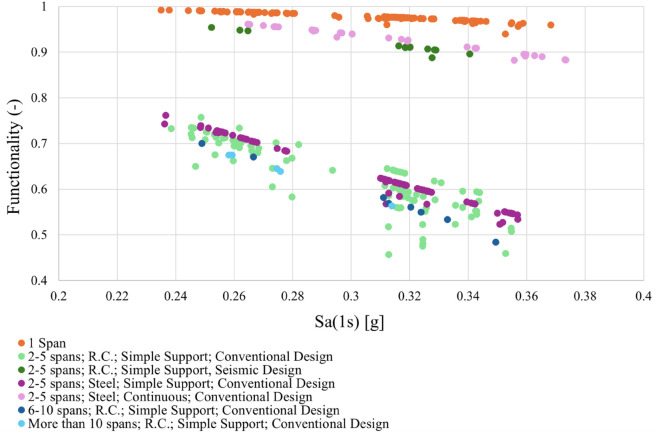
Functionality of the considered bridges as a function of the spectral amplitude, for different construction materials, structures, and seismic design prescriptions.

In the considered case study, 181 bridges have a functionality level between 75% and 100%, 182 bridges between 50% and 75%, and 6 bridges between 25% and 50% (Fig 5). These findings indicate that many bridges in Foggia province are not classified as high-risk for seismic failure, reflecting their resilience during past seismic events. As shown in Fig 6, the functionality level decreases, i.e., the bridge’s performance worsens, as the seismic demand’s spectral amplitude increases. The graph also illustrates how the functionality of bridges, as derived from the proposed fragility model, is influenced by their constructive and structural features. For instance, one-span bridges consistently exhibit the highest functionality, with values never falling below 90%, which aligns with research indicating their generally lower vulnerability to seismic actions compared to multi-span structures [44]. Conversely, Fig 6 clearly shows a decrease in functionality as the number of spans increases, a trend consistent with studies highlighting the heightened vulnerability of multi-span bridges [45,46]. Furthermore, the results demonstrate that reinforced concrete bridges designed following seismic regulations are significantly less susceptible to damage than those without such provisions [47]. Lastly, the graph indicates that steel bridges with simply supported decks are more vulnerable than those with continuous decks. This observation is supported by literature, which identifies multi-span steel girder bridges with simple supports as among the most vulnerable bridge types [44]. Studies consistently show that simply supported deck bridges, particularly older designs, are more susceptible to seismic damage and exhibit lower resistance to earthquake intensities compared to their continuous deck counterparts [46,48,49].

### The bridge criticality score

**Fig 7:**
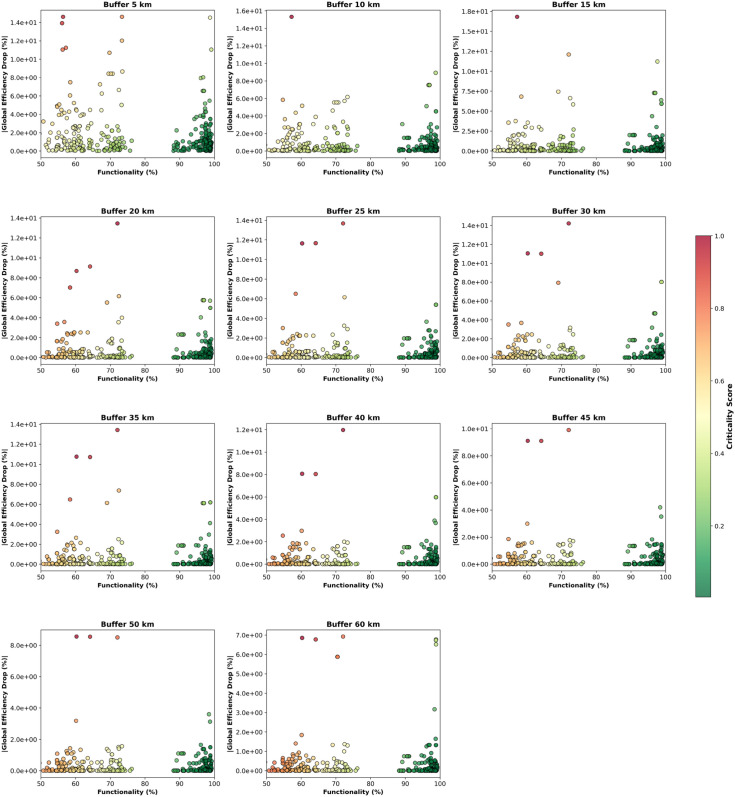
Criticality score of bridges, with the systemic impact on the road network evaluated at different distances from the disruption. Each graph includes a scatterplot where the horizontal axis represents the bridge’s functionality, and the vertical axis represents the absolute value of the efficiency loss % after its disruption, evaluated at the considered distance. Points correspond to individual bridges and are colored according to the criticality score. The color coding highlights the distribution of the criticality values across the plot, providing an immediate visual cue for bridges with higher (top left region in the plot) or lower (bottom right) criticality scores.

This study introduces a bridge criticality score, defined as the average of the normalized efficiency drop and the normalized functionality loss in case of disruption (see Materials and Methods for a detailed definition). This quantity ranges between 0 (low criticality) and 1 (high criticality) and depends on the distance from the bridge at which the road network efficiency drop is computed. Fig 7 shows the bridge criticality score for all bridges analyzed and comprises different panels referred to varying distance ranges used in evaluating the road network efficiency drop. Each panel displays a scatterplot, where the horizontal axis represents the bridge’s functionality, and the vertical axis represents the absolute value of the efficiency drop percentage evaluated at the considered distance from the disruption. Points correspond to bridges, and their color gradient visually conveys the values of the criticality score, ranging from 0 (green) to 1 (red). This framework allows pinpointing the most critical bridges, exhibiting at the same time low functionality and a relevant systemic impact on the surrounding road network in case of disruption.

The analysis identifies the most critical bridge at 41.587241^°^N, 15.507174^°^E, located on the SP22 tertiary road. This bridge shows the highest criticality score (0.892) at radii of 5, 10, and 15 km, indicating its key role at local scales. The bridge located at coordinates 41.866273^°^N, 15.664665^°^E, along the SS693 trunk road, exhibits a criticality score of 0.761 on circular areas with radii between 20 and 30 km from the disruption. Another pivotal bridge has been identified at 41.836819^°^N, 15.734399^°^E, on the same SS693 trunk road. Its criticality score increases with the distance from the disruption and ranks first for areas with radii between 35 km (score 0.794) and 60 km (0.864), highlighting its regional significance. The bridges located at 41.897113^°^N, 15.257009^°^E, and at 41.896939^°^N, 15.256894^°^E, both on the primary A14 road, have criticality scores appearing in the top ten for all the considered buffer distances around the disruption. The bridge located at 41.896911^°^N, 15.257279^°^E, on the A14 primary road, is among the 10 most critical ones for 10 different distances, with scores ranging from 0.52 to 0.66.

### Impact of critical bridge disruptions on inner area municipalities

**Fig 8:**
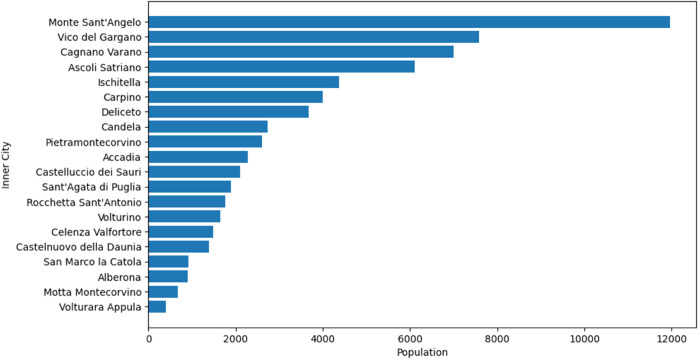
Population distribution of inner municipalities at risk of isolation.

**Fig 9:**
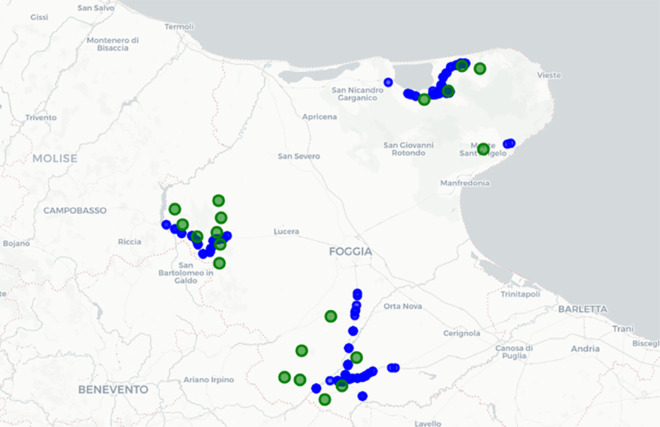
Inner municipalities within bridge buffer zones. Green circles represent inner municipalities, while blue circles indicate bridges. This map is generated using the Folium open-source library, which uses OpenStreetMap as a map source [50].

Inner municipalities, typically rural and sparsely populated, often have limited access to essential services and are particularly vulnerable to disruptions in the transportation network due to their geographical isolation. To quantify this vulnerability, the study integrates demographic inner municipality data with a topological analysis of the road network, identifying which inner municipalities face the highest risk of isolation after bridge failures. The analysis focuses on 34 municipalities in the Foggia province, that are officially classified as “inner area municipalities”, based on criteria such as low population density and limited alternative routes. This classification is drawn from the official list of Italian inner areas, ensuring selection is based on recognized, objective criteria (see Materials and Methods section). Among these 34 inner area municipalities, 20 are located within the buffer zones of bridges, defined as the areas around each given bridge where the variation of relative efficiency drop with the radius is relevant (a quantitative definition is reported in Materials and Methods). The study thus identifies these 20 inner municipalities as high-risk. Out of 369 bridges considered in this study, 79 are crucial for preserving inner areas’ connectivity. The potential failure of these bridges threatens to isolate 57,930 residents, causing significant disruptions to regional connectivity. Fig 8 shows the population distribution of inner municipalities facing this isolation risk. The spatial distribution of bridges and inner municipalities within their critical buffer zones is illustrated in Fig 9.

Among the 79 crucial bridges for inner-municipality connectivity, several stand out for their extensive reach across various inner municipalities within their buffer areas. These bridges act as vital links, and their disruption can disproportionately affect regional connectivity. To evaluate the impact of bridge removal on the connectivity of an inner area municipality, inner nodes are first defined as those located within its administrative boundaries, determined using ISTAT shapefiles [51]. The analysis then focuses on peripheral nodes, a crucial subset of inner nodes defined as those within the municipal boundary that maintain at least one direct connection to an external node. S1 Fig in S1 File illustrates this distinction for the inner area municipality of Alberona. This restriction to peripheral nodes reduces the total node count by approximately 95% while effectively capturing all critical entry and exit points for each municipality. The study measures the closeness centrality of peripheral nodes situated within the critical buffer zones of bridges. The dataset includes 79 bridges with at least one inner area municipality in their buffer zones, and 20 inner municipalities located within at least one bridge buffer zone. This overlap yields 217 unique bridge-inner municipality pairs for which data is collected. Measuring the change in closeness centrality for all affected peripheral nodes generates over 7300 data points, enabling a comprehensive analysis of results from three distinct perspectives, as detailed subsequently.

#### Aggregated level.

The impact of bridge disruptions on inner-area municipalities is first measured at an *aggregated level*, namely focusing on the joint set of peripheral nodes that connect urban centers. Within this framework, each bridge is associated with the inner municipalities located in its buffer zone, and the analysis quantifies its impact on surrounding connectivity by averaging the change in closeness centrality across the municipalities’ peripheral nodes.

Results show that the removal of many minor bridges, particularly those on tracks (crossing major routes like the A16, E842, and SS665), generates little to no impact on the overall average closeness change of all municipalities lying within the bridge buffer zone. In contrast, removing bridges on trunks and motorways leads to a measurable average decline in the closeness of peripheral nodes of municipalities in the bridge buffer zone. The outcomes suggest that minor roads typically provide alternative paths and redundancy, and hence, the network structure can absorb their loss. This interpretation is supported by Berche et al., who demonstrated that networks with greater redundancy and clustering – attributes characteristic of systems with numerous minor connections and loops – exhibit stronger resilience to targeted failures [52]. Their findings confirm that structural redundancy is critical for maintaining connectivity in regional transport networks and highlight the strategic value of alternative routes in planning for resilience [36]. Table 1 summarizes bridges whose removal determines the most pronounced impact on inner-area connectivity; it is worth highlighting that all of them are on the SS693 trunk road.

**Table 1:** Top bridges ranked by their aggregated-level impact on the connectivity of inner area municipalities. For each bridge, the table also lists the most affected municipalities, alongside their average closeness centrality drop, quantified as the average drop of peripheral nodes.

Bridge Coordinates	Inner Municipality	Avg. Closeness Drop %
41.8786^°^ N 15.8398^°^ E	Ischitella	4.66
	Carpino	3.66
	Vico del Gargano	1.42
	Cagnano Varano	0.99
41.8802^°^ N 15.8421^°^ E	Ischitella	4.60
	Carpino	3.62
	Vico del Gargano	1.40
	Cagnano Varano	0.98
41.8362^°^ N 15.8068^°^ E	Carpino	7.16
	Ischitella	5.96
	Cagnano Varano	1.00

#### Inner-municipality level.

The second approach aims at quantifying the effects of bridge disruptions at the *inner-municipality level*, by measuring the impact of a single bridge removal on a specific municipality within its buffer zone as the average closeness centrality drop, computed over the set of the municipality’s peripheral nodes. The analysis, performed for the 217 bridge-municipality pairs of interest, highlights variations in local accessibility resulting from targeted disruptions. While the closeness average change at the aggregated level is generally minor, certain municipalities exhibit considerably higher sensitivity. Notably, Cagnano Varano, Carpino, and Ischitella undergo the largest closeness average changes, suggesting greater exposure to isolation driven by specific bridge disruptions. Specifically, the removal of the SP41 trunk bridge at coordinates 41.914164^°^N, 15.673086^°^E results in a 24.07% average decrease in the closeness centrality of Cagnano Varano peripheral nodes. According to OpenStreetMap data, this bridge spans a water canal, underscoring its strategic role in maintaining local connectivity. A relevant effect is also observed for Carpino, where eliminating the SS89 trunk bridge at 41.857250^°^N, 15.814833^°^E leads to a 7.33% average reduction in the closeness of peripheral nodes. This result is consistent with the critical role of the SS89 – part of a major state road linking towns such as Vieste, Peschici, and Rodi – in the regional transport network [10]. As concerns Ischitella, the removal of the trunk bridge on the SS693 at coordinates 41.836278^°^N, 15.806750^°^E causes an average decrease of 5.96% in the closeness centrality of peripheral nodes; the same bridge also emerges among the most critical ones for inner area connectivity according to the aggregated-level analysis (see Table 1). The SS693 state road provides key access across the northern Gargano region, further emphasizing the local impact of its disruption [53].

These average changes, computed across all the peripheral nodes of a municipality within the buffer radius of a bridge, for all the 217 bridge-municipality pairs, demonstrate that the systemic role of bridges is highly context dependent. Bridges that serve as primary connectors, especially those that enclose natural barriers, typically exert a more pronounced influence on the structure and accessibility of the network when removed. The higher impact observed for the SP41 bridge on the average closeness centrality of Cagnano Varano (24.07% decrease) compared to the SS89 bridge for Carpino (7.33% decrease) and the SS693 bridge for Ischitella (5.96% decrease) may indicate that SP41 functions as a more unique or less redundant link in its surrounding network. In contrast, SS89 and SS693 may offer more alternative routes or distribute their connectivity benefits over a broader area. These differences reflect how network topology and redundancy shape the criticality of individual elements of the road infrastructure.

#### Peripheral-node level.

Finally, the study evaluates the effects of bridge disruptions on the isolation of inner areas by capturing the closeness centrality drop for each municipality peripheral node within the buffer zone of the bridge under investigation. This *peripheral-node level* assessment enables the identification of localized disruptions in accessibility, indicating whether the removal of a given bridge alters the connectivity of the corresponding municipality to surrounding areas through specific road segments. The most substantial change is observed in Candela, where the removal of a motorway bridge on the E842/A16 road at coordinates 41.148333^°^N, 15.507999^°^E results in a 99.42% decrease in closeness centrality for a peripheral node located on SP101 (41.148639^°^N, 15.506754^°^E). This bridge passes over SP101, which serves as a key internal–external connection for the municipality. The near-total drop in closeness indicates a critical reliance on this bridge for maintaining accessibility, with limited redundancy in the nearby network.

#### Comparison with a null model.

To assess whether the observed changes in closeness centrality at the peripheral-node level reflect a structural behavior of the road network that significantly differs from random chance, the results are compared against a null hypothesis framework. This involves generating randomized graphs that preserve the degree distribution of the original subnetworks [54,55] (further details on the configuration model and randomization are provided in the Materials and Methods).

For each of the 79 targeted bridge removals, the accessibility loss (quantified as the closeness centrality drop for peripheral nodes) is compared with the distribution of losses from random failures within the corresponding null model using the Wilcoxon signed-rank test [56]. The distribution of p-values obtained from this analysis across all 79 bridges yields a median p-value of 0.006. This low median value provides strong evidence to reject the null hypothesis for the majority of cases, confirming that the observed accessibility losses caused by most bridge failures are structurally determined rather than being a result of random chance. The mean p-value of 0.081, while higher, is skewed by a subset of non-critical bridges whose impacts are statistically indistinguishable from those generated by random failures in the null model. This highlights the heterogeneous vulnerability within the network, where some bridges are indeed critical due to their structural position, while others are not. Overall, the consistently low median p-value underscores that the observed closeness centrality drops are a characteristic structural property of the road network.

## Discussion

The present work adopts a complex network approach to quantify the impact of bridge failures on road network performance at different distance scales, and combines it with structural vulnerability evaluations to define a bridge criticality score. This network-wide integrated framework aligns with established seismic-fragility and resilience research. Specifically, Borzi et al. developed fragility curves for over 17000 bridges and integrated them into GIS-routing models [57], while Capacci et al. used life-cycle probabilistic frameworks to illustrate how aging, design, and redundancy govern bridge criticality [58]. Moreover, the proposed methodology allows for an assessment of the isolation risk related to inner municipalities, unveiling the fragility of their road connections. The obtained results constitute a potential basis for developing optimization models to prioritize bridge retrofitting and maintenance [19], combining information on individual bridge vulnerabilities and an evaluation of how their failure would impact at the network level. The proposed framework highlights the role of bridge functionality and strategic placement in assessing road network resilience. The bridge criticality analysis has identified low-functionality assets whose failures cause significant spillover effects on network efficiency across multiple buffer distances, highlighting localized effects and broader impacts. Importantly, the developed novel algorithm based on the definition of a buffer distance can be easily scaled up to national or larger levels, thanks to its inherent scalability.

The analysis highlights several key infrastructure elements that play a crucial role in network resilience across different spatial distances. At local scales (5–15 km), a bridge situated on the SP22 tertiary road exhibits the highest criticality, emphasizing its importance in short-range connectivity. As the distance from the disruption increases (20–30 km), a bridge on the SS693 trunk road shows similar vulnerability patterns, with a criticality score of 0.761, suggesting its role in sustaining medium-range paths. At the province scale (35–60 km), another bridge of the SS693 trunk road becomes increasingly critical, with its importance growing at larger distances. Interestingly, Furinghetti et al. performed fragility analyses on aging concrete structures comparable to those on SP22 and SS693, reinforcing the identification of these corridors as highly vulnerable [59]. Moreover, two bridges on the A14 primary road consistently rank among the most critical ones across the considered radii, highlighting their significance at multiple spatial scales. These findings illustrate how bridge disruptions affect road network connectivity differently, demonstrating the sensitivity of the proposed framework to identify critical assets, differentiating between localized bottlenecks and structurally central routes.

Bridge failures do not affect the accessibility of inner municipalities in a uniform way. By analyzing the results across three levels (aggregated, inner-municipality, and peripheral-node), this study offers a more detailed understanding of how bridge disruptions influence municipal connectivity. At the aggregated level, many bridges, especially those located on minor roads or tracks, show little to no effect on the average accessibility of nearby municipalities. Such a result highlights the important role of network redundancy, which allows the system to absorb certain failures without significant disruption. These findings are consistent with theoretical models suggesting that peripheral links, while not central in daily operations, play a hidden but crucial role in maintaining connectivity during failures [60]. The low impact of removing minor bridges also reflects a spatial structure where multiple alternative connections are available, even if they are not significantly used until needed. In contrast, the removal of bridges on trunk roads and motorways leads to clear drops in the average closeness centrality of affected municipalities. These higher-tier roads are designed to concentrate traffic flow, which makes them efficient under normal conditions but also vulnerable to disruption. Their failure tends to affect a wider area, reducing accessibility for several municipalities at once, a pattern often seen in networks with centralized or hub-like structures [14].

Zooming in to the inner-municipality level, the effects of bridge removal become more varied. Some municipalities experience a significant loss of accessibility, while others remain largely unaffected. This difference often depends on the municipality’s position with respect to the disrupted bridge, and on how much it relies on that specific connection. For example, Cagnano Varano and Carpino appear particularly vulnerable, not because they lack infrastructure overall, but because key bridges serve as main access points linking them to the regional network. In these cases, even considering the redundancy of the regional network, the local impact can be severe. This suggests that evaluating the importance of each bridge should go beyond its general role in the network and also consider how it affects individual municipalities. Remarkably, the findings regarding the high risk of isolation for Cagnano Varano and Carpino due to bridge inaccessibility are strongly corroborated by real-world events: the 2016–2017 Central Italy earthquakes, for instance, witnessed analogous bridge failures that led to critical disconnections [61], mirroring the network impacts modeled in the proposed framework for these inner municipalities.

The peripheral-node level analysis reveals specific weaknesses that are not visible at coarser scales. In Candela, for instance, the removal of a motorway bridge results in a dramatic drop (over 99%) in the closeness centrality of a single peripheral node that connects the town to the broader road network. Although the municipal average of the closeness centrality drop may not reflect such an extreme case, this example shows how certain nodes can be critically dependent on a single bridge. These highly localized vulnerabilities often relate to how the road network is physically structured, such as where roads cross or where terrain limits the placement of additional links.

Together, findings at the three levels of the analysis emphasize the need for resilience planning to operate towards multiple targets: ensuring regional redundancy, protecting key municipal connectors, and identifying critical nodes where single failures can cause major disruptions. Such observations are supported by the works of Rasulo et al., in which time-dependent resilience metrics were adopted to quantify the reduction in the network capacity and overall functionality following an earthquake, demonstrating that isolated municipalities suffer more severe accessibility losses upon bridge failure [62,63].

Finally, null hypothesis testing via randomized configuration models demonstrates that the disruptions in peripheral node centrality observed in the proposed framework are not statistical artifacts, but reflect the genuine topological importance of specific bridges. The consistently low p-values across most bridges support rejecting the null hypothesis, indicating that the closeness centrality drops of peripheral nodes in inner area municipalities significantly differ from those generated in the configuration model framework. This result confirms that targeted bridge failures have a stronger impact on the isolation of vulnerable communities than random failures [14].

## Conclusion

The proposed framework provides the basis to develop a decision support tool that can assist infrastructure managers in prioritizing resource allocations, planning target interventions, and defining customized backup plans for various road types and locations, thus enhancing infrastructure resilience and disaster preparedness.

Despite the comprehensive approach of this study, several limitations should be acknowledged. First, the bridge criticality score combines the two component indicators, F^*^ and D^*^(*R*), using a simple arithmetic mean with equal weighting. The authors recognize that the relative importance of functionality versus network impact can change depending on specific situations, such as during emergencies or in long-term planning. For instance, post-disaster response might prioritize network connectivity (D^*^(*R*)), while long-term planning could focus more on bridge functionality (F^*^). While the model discussed in this article offers a general, balanced measure, a promising area for future research involves developing adaptive weighting schemes. This would enable more precise, policy-driven prioritization of bridges, moving beyond a single, fixed “criticality” score to one that can be customized for specific goals or changing operational needs. Another key aspect is that the model does not currently account for real-world traffic patterns. Actually, integrating the analysis with traffic flow simulations reproducing, e.g., the dynamic redistribution of vehicles after bridge disruption, can enhance the definition of emergency response strategies. Moreover, while the proposed framework quantifies the impact of bridge failures, it overlooks the potential disruptions to underlying street segments. Future research should leverage the information available on infrastructures [30], including data from structural health monitoring systems, to calibrate and update fragility curves based on real-time bridge performance, highlighting degradation of functionality due to aftershocks and long-term damage. Building upon this, a significant aspect for more refined analyses is the nonlinear Soil–Structure Interaction (SSI). SSI is crucial for seismic performance-based design of bridges [64] and other critical infrastructures, such as nuclear power plants [65] and overhead transmission lines [66], as it accurately captures the complex dynamic behavior at the soil-structure interface during earthquakes [67,68]. Incorporating SSI, typically via three-dimensional finite-element models and nonlinear time history analyses for time-variant fragility curves [33], would provide more accurate evaluations of bridge response, differentiating vulnerability by type and geometry [69] and integrating aging effects [34]. To fully implement such an approach, comprehensive site-specific geotechnical data and more computationally intensive numerical models are necessary.

In general, future research can benefit from integrating machine learning algorithms in the workflow, exploiting large datasets of seismic events and bridge damage to improve the accuracy of functionality predictions, fragility curves, and critical bridge identification [70]. Moreover, developing multihazard assessment frameworks that consider the combined effects of seismic events along with other natural hazards [71], such as flooding or landslides, would enhance the robustness of the analysis. Expanding the study to include socioeconomic factors and critical facility accessibility [2] would also provide a multifaceted understanding of network resilience, supporting more equitable and informed infrastructure investment strategies. Finally, the proposed methodology could be further developed for immediate application following an actual seismic event to assess bridge damage and its potential impact on the road network.

## Materials and methods

### Road network construction

The road network data for Apulia, Italy, has been extracted from OpenStreetMap (OSM) in GeoJSON format [53]. The dataset includes road segments, classified hierarchically into 12 types, following the OSM highway tagging system (Table 2) [53], with specific labels for bridge segments. Geometries have been projected onto a Coordinate Reference System (CRS) to ensure spatial accuracy. The focus is on the towns in Foggia province that are classified as inner area municipalities because of their low population density and limited access to services, according to Italy’s Partnership Agreement 2014–2020 within the National Strategy for Inner Areas (SNAI) [25]. The shapefiles defining the borders of these areas have been sourced from ISTAT [72]. Although the road network covers the entire Apulia region, the focus on vulnerable bridges is specific to Foggia province. Open-source Python libraries such as Pandas [73], GeoPandas [74], Geopy [75], Folium [50], and NetworkX [76] have been used for data processing, map generation, and the computation of topological indicators.

**Table 2:** Description of the road type labels used by OSM, ordered from most important (motorway) to least important (track).

Road type	Description
Motorway	Provides high-speed, limited-access
	roads for long-distance travel.
Motorway Link	Connects a motorway to another road type.
Trunk	Serves as major national/regional roads that are
	wider than primary roads.
Trunk Link	Connects the trunk road to another road.
Primary	Connects towns and cities, handling high traffic volume.
Primary Link	Connects the primary road to other road types.
Secondary	Connects towns and villages,
	less important than primary roads.
Tertiary	Links smaller settlements or connects to larger roads.
Unclassified	Provides minor roads in rural or less developed areas.
Residential	Provides access to housing areas.
Services	Provide access to properties or businesses.
Track	Serves agricultural/forestry use, often as an unpaved road.

Graph theory provides a structured framework for analyzing a road network, represented as an undirected graph G=(V,L), with *V* denoting the set of nodes and *L* the set of links. The connectivity of the network is captured by the adjacency matrix *A* = [*a*_*ij*_*]*, where:


$$a_{ij}=\left\{ \begin{array}{cc} 1, & \text{if there is an edge between nodes }i\text{ and }j\\ 0, & \text{if there is no edge between nodes }i\text{ and }j. \end{array}\right.$$

**Fig 10:**
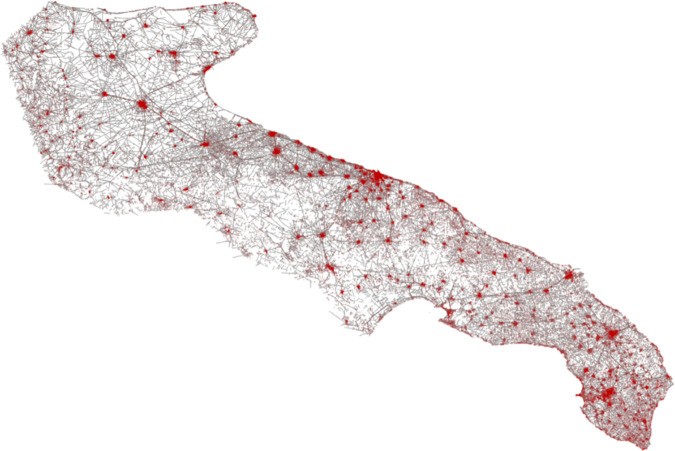
Graph representation of the Apulian road network illustrating nodes as road LineString endpoints and links as roads. The map is generated using the Folium open source library, which uses OpenStreetMap as a map source [50].

The matrix *A* represents the structure of the graph *G* in a compact form; for an undirected graph, the matrix is symmetric, meaning $a_{ij}=a_{ji}$. To construct the road network graph *G*, geospatial road data stored in a GeoData frame have been processed using the NetworkX library. Each road segment is represented as a geometric object, either a LineString or MultiLineString, from which the start and end coordinates are extracted. Formally, for each road segment *e*_*k*_, the start and end points are defined as:

$$e_{k}=(n_{i},n_{j}),\;n_{i},n_{j}\in V,$$ (2)

where $n_{i}=(x_{i},y_{i})$ and $n_{j}=(x_{j},y_{j})$ are the geographic coordinates of the nodes. Nodes are uniquely defined at road segment endpoints, ensuring a consistent representation of intersections and terminal points. Bridges are identified as a subset B⊆L based on their attributes, and their disruption is simulated by removing the corresponding edges from *G*. The validation step ensures that all geometries are well-defined and non-empty before constructing the final network. The resulting graph integrates the nodes, edges, and attributes of the road, including the status of the bridge, facilitating further network analysis [60]. In Fig 10, red points represent nodes (n=95919), and gray lines represent road segments as links (n=65017) within the Apulian road network.

### Resilience of the road network under targeted bridge removal

The systemic vulnerability of a network is evaluated by assessing the impact of bridge removals, which can isolate regions and disrupt connectivity [77]. Targeted removal scenarios allow us to analyze the road network’s resilience to strategic attacks. This study systematically removes individual bridges, measuring changes in network metrics to quantify the effects of these failures on connectivity [78,79]. A road network’s resilience relies on its topology, with critical elements assessed for their role in maintaining connectivity. Key indicators like average path length and global efficiency provide insights into network structure and resilience [80].

#### Global efficiency.

Global efficiency $E_{\text{glob}}$ quantifies the network’s ability to maintain connectivity by considering the inverse of shortest path lengths between nodes. It is defined as [81]:

$$E_{\text{glob}}=\frac{1}{N(N-1)}\sum_{i\neq j}\frac{1}{d_{ij}}$$ (3)

where *N* is the total number of nodes and *d*_*ij*_ is the shortest path length between nodes *i* and *j*. If no path exists between two nodes, *d*_*ij*_ is considered to be infinite. Higher global efficiency indicates a more resilient and well-connected network, even under disruptions [82].

#### Edge betweenness centrality.

Edge betweenness centrality *C*_*B*_(*e*) quantifies the importance of an edge *e* in the network by measuring how frequently it appears on the shortest paths between all pairs of nodes. It is defined as [83]:

$$C_{B}(e)=\sum_{s\neq t}\frac{\sigma_{st}(e)}{\sigma_{st}}$$ (4)

where $\sigma_{st}$ is the total number of shortest paths between nodes *s* and *t*, and $\sigma_{st}(e)$ is the number of those paths that pass through edge *e*. Edges with high betweenness centrality are critical for efficient communication and flow within the network and are often key to understanding network vulnerability.

### Functionality of bridges

Estimated seismic hazard data have been obtained from the Istituto Nazionale di Geofisica e Vulcanologia (INGV) and the Italian seismic code for structural design (NTC18) [43]. The 50th percentile Peak Ground Acceleration (PGA) values, corresponding to a return period of 475 years (0.004 annual exceedance probability, that is, 10% chance over 50 years), have been extracted from the MPS04-S1 seismic hazard model using the Centro di Pericolosità Sismica (CPS) web platform and bridge coordinates [84]. The elastic acceleration spectrum for each bridge, used as input for the fragility curves, has been obtained following the prescription of NTC18 [43]. The subsoil category has been derived from the average shear wave velocity in the top 30 meters obtained from [85]. At the same time, the slope has been calculated based on the Digital Elevation Model (DEM) provided by INGV [86]. Due to the preliminary scope of this study and the absence of detailed site-specific data for each bridge, explicit consideration of soil-structure interaction effects was not feasible. This framework has allowed us to conduct a broad assessment across a larger inventory of bridges, focusing primarily on the structural performance of the bridge elements themselves.

**Fig 11:**
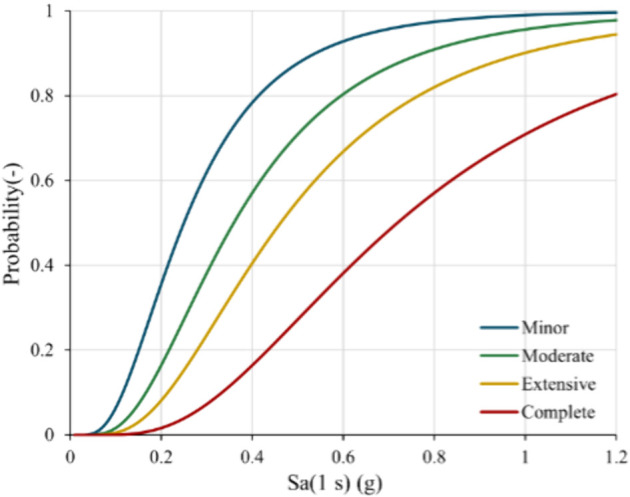
Fragility curves of a bridge for different damage states. The horizontal and vertical axes represent, respectively, the amplitude *Sa*(1*s*) of the spectral acceleration at 1.0 seconds, and the conditional probability of reaching and/or exceeding a specific damage state (minor, moderate, extensive, complete).

The empirical fragility curves developed in the RISK-UE project [31] have been employed to assess the vulnerability of the bridges of interest and estimate their functionality in the event they are affected by an earthquake with a return period of 475 years. The approach categorizes bridges into fifteen classes based on material, span continuity, type of piers, and use of seismic design prescription. Then, for each class, using parameters that consider the number of spans, the skew of the bridge, and the earthquake features, fragility curves are obtained for four damage states: minor, moderate, extensive, and complete (Fig 11). The fragility curves depend on the spectral acceleration at 1.0 seconds Sa(1s), which is used as an input parameter for estimating the probability of reaching or exceeding the considered damage states. The intensity measure Sa(1s) is widely adopted for bridge fragility assessments due to its effectiveness in capturing seismic energy relevant to typical bridge periods and its practicality for broad portfolio analyses [87]. The data relative to the structural parameters of the bridges are the result of mixed techniques and sources, which will be expanded and refined in future work.

Finally, by assigning a functionality level to each damage state, knowing their probability to be reached, the overall functionality level (F) of each bridge has been derived using the following formula:

$$F=\sum_{i}P_{i}F_{i},$$ (5)

where *P*_*i*_ is the probability of reaching a damage state higher than *i* and not exceeding i+1, and *F*_*i*_ is the functionality level assigned to the same damage state. It is worth noticing that the functionality, as derived, summarizes information about structural vulnerability and seismic hazard.

### Combining efficiency and functionality losses in a bridge criticality score

To prioritize bridges based on their functionality and systemic impact on the road network, this research introduces a combined criticality score. This metric integrates two key factors: the relative efficiency drop at distance *R* from the bridge *D*(*R*) in Eq 1 and the bridge functionality *F* in Eq 5, ensuring that structures with higher network impact and lower functionality values are ranked as more critical. The criticality score is computed through a two-step normalization process. To make functionality and global efficiency drop values comparable, this study defines the normalized functionality loss as

$$F^{*}=1-\frac{F-\min(F)}{\max(F)-\min(F)}\;,$$ (6)

with max(F) and min(F), the maximum and minimum functionality evaluated on the set of all bridges, and the normalized absolute efficiency drop

$$D^{*}(R)=\frac{\left|D(R)|-\min(|D|\right)}{\max(|D|)-\min(|D|)}\;,$$ (7)

with max(|D|) and min(|D|), the maximum and minimum absolute value of the relative efficiency drop evaluated on the set of all bridges for all the distances considered in the present study. The normalized values are averaged to obtain the criticality score

$$C(R)=\frac{F^{*}+D^{*}(R)}{2},$$ (8)

providing a balanced measure of functionality loss and network impact, which enables effective prioritization of critical bridges.

F^*^ and D^*^(*R*) offer largely distinct yet complementary perspectives on bridge criticality. While F^*^ captures an asset’s intrinsic structural vulnerability, D^*^(*R*) quantifies the systemic impact of its potential failure on network connectivity. As these indicators are conceptually independent, combining them in a criticality score allows for a comprehensive assessment of road network vulnerability from multiple angles. The use of an equal-weight linear combination in Eq (8) ensures neither the bridge’s internal condition (F^*^) nor its network impact (D^*^(*R*)) unfairly dominates the assessment. This balanced approach, common in early modeling stages for its clarity [88,89], provides a solid foundation for prioritizing bridges by equally valuing their structural vulnerability and importance to the surrounding road network. Consequently, a bridge is flagged as critical if it is either in poor condition or vital to the network, offering a comprehensive and transparent pilot ranking for risk management.

### Resilience of inner-area municipalities against bridge disruptions

Italy’s Inner Areas, characterized by geographical remoteness and reliance on road infrastructure, are critical for the nation’s territorial balance and sustainability. These sparsely populated regions are highly vulnerable to connectivity disruptions, such as bridge failures during natural hazards. Classified under the National Strategy for Inner Areas (SNAI) [25], they face isolation from essential services like healthcare and education, making them susceptible to infrastructure failures. To accurately assess these vulnerabilities, the SNAI framework categorizes municipalities by their level of internality, considering factors like proximity to service centers, socioeconomic conditions, and demographic trends. Inner municipalities within these areas are classified as intermediate, peripheral, or ultra-peripheral, with the latter facing the highest risk due to extreme isolation.

This research employs a spatial analysis approach to identify bridges whose failure could exacerbate isolation risks in inner municipalities. Rather than conducting computationally intensive network analyses for every bridge failure scenario, the focus is on the network segments enclosed in a circular area of varying radius *R* centered on each bridge, to capture the impact of disruptions across different distance scales. Specifically, a municipality *u* is considered inside the area around a bridge *b* if

d(u,b)≤R, (9)

where *d*(*u*,*b*) denotes the Euclidean distance between the geographical locations of the municipality center and the bridge, whose spatial coordinates have been collected from Geopy [75] and OSM [53], respectively.

#### Identifying bridge’s high-risk zones.

For each bridge, this analysis defines the buffer radius as $R_{\text{buffer}}$, representing the range in which the variation of global efficiency drop is most relevant, through an iterative tuning process. First, the change in global efficiency across incremental radii, starting from 5 km up to 50 km in 5 km steps, is measured, followed by an additional measurement at 60 km to account for broader network effects. This range enables one to detect both highly localized and more widespread impacts without incurring excessive computational overhead. At each step, it computes reduction in global efficiency $\Delta E_{\text{glob}}(R)$ is computed. Formally, the buffer radius is defined as the smallest value of *R* such that

$$\left|\frac{\Delta E_{\text{glob}}\left(R\right)-\Delta E_{\text{glob}}\left(R+\delta R\right)}{\Delta E_{\text{glob}}\left(R\right)}\right|\leq 40\%,\;\text{where }\;\delta R=\left\{ \begin{array}{cc} 5\text{ km}, & R<50\text{ km}\\ 10\text{ km}, & R=50\text{ km} \end{array}\right.$$ (10)

with $\Delta E_{\text{glob}}(R)=E_{\text{glob}}(R)\;-\;E_{\text{glob}}^{0}(R)$, the difference between global efficiency after bridge removal and before. This condition ensures that $R_{\text{buffer}}$ effectively captures the impact of bridge failure while avoiding unnecessary computational overhead. The study iteratively expands buffer radii until the reduction in global efficiency stabilizes, ensuring that each zone accurately reflects the bridge’s impact on network connectivity. The 40% cutoff value has been identified based on consistent patterns observed across all bridges. For over 80% of the considered bridges, the relative change in $\Delta E_{glob}(R)$ between consecutive radii stabilizes once it drops below 40%, indicating that further increases in radius yield no significant additional changes. For each bridge, this threshold defines a buffer radius that effectively encompass the most influential spatial extent of a possible failure, providing a practical and interpretable way to define the bridge’s area of influence while avoiding excessive computational effort and inclusion of marginal effects.

#### Peripheral nodes of inner area municipalities.

For each inner area municipality, the shapefiles representing their administrative boundaries are collected. Nodes located within these boundaries are defined as *inner nodes*. Among these, the analysis identifies the crucial subset of *peripheral nodes*, defined as the inner nodes connected by a road segment (link) to at least one node external to the municipality’s border. The figure in S1 File provides a map of the inner area municipality of Alberona, illustrating the distinction between peripheral and non-peripheral inner nodes. Peripheral nodes function as critical gateways, channeling all incoming and outgoing traffic and thus linking the internal urban network to the broader inter-municipality road system. To effectively evaluate the impact of bridge failure on the connectivity of the inner area municipalities, the analysis is focused on peripheral nodes, since a municipality’s inbound and outbound accessibility is entirely mediated through these transition points. Specifically, the impact of accessibility loss on inner area municipalities is quantified by evaluating the change in closeness centrality of these peripheral nodes following bridge removal, as detailed in the subsequent section.

#### Closeness centrality of peripheral nodes.

Closeness centrality quantifies a node’s accessibility within a network, indicating how efficiently it can reach all other nodes. In road networks, nodes with higher closeness centrality can access a larger portion of the network with fewer steps, reflecting superior connectivity [90]. Mathematically, the closeness centrality *C*_*i*_ of a node *i* is defined as:

$$C_{i}=\frac{N-1}{\sum_{j\in V}d_{ij}},$$ (11)

where *N* is the total number of nodes in the network, *d*_*ij*_ represents the shortest path distance between nodes *i* and *j*, and *V* is the set of all nodes in the graph [82].

This study uses closeness centrality to evaluate the connectivity of the peripheral nodes of inner municipalities before and after the removal of selected bridges. A decrease in closeness centrality following bridge removal indicates reduced accessibility, potentially isolating certain regions and highlighting critical infrastructure bridges that require prioritization for maintenance or reinforcement. To determine the impact of bridge removal on the accessibility of the most isolated and vulnerable areas, the research examines the reduction in closeness centrality of peripheral nodes across three distinct scales: first, at an *aggregated level*, where the study considers the average closeness drop across all the peripheral nodes of municipalities within the bridge’s buffer zone; second, at the *inner-municipality level*, where the reference indicator is the average closeness drop across all peripheral nodes of a given municipality; and third, at the *peripheral-node level*, where reductions in closeness are examined separately for each individual node. Applying these three approaches conjointly provides complementary insights into the multifaceted impact of bridge failures on network accessibility.

#### Null hypothesis benchmarking.

Benchmarking network disruptions using configuration models is a well-established approach in network science. The configuration model, first formalized by Bollobás [91] and further popularized by Newman [92], provides a null framework for assessing whether empirical network properties significantly deviate from random chance expectations given degree constraints. A null hypothesis framework based on a configuration model [54,55] is developed to evaluate whether peripheral-node-level changes in closeness centrality of inner-area municipalities reflect a structural behavior of the road network that differs significantly from that of a random graph. For each of the 79 bridges, 100 randomized graphs are generated from the original subnetwork restricted to the bridge’s buffer area, with the constraint of preserving its degree distribution. The closeness centrality of peripheral nodes of inner municipalities is calculated before and after bridge removal, and the relative change is measured. This procedure is repeated for all the over 7300 bridge–peripheral node pairs of interest. For each targeted bridge removal, the analysis compares the accessibility loss, quantified as the closeness centrality drop of municipality peripheral nodes within the bridge buffer radius, against the distribution of losses from random failures in the null model. The statistical comparison is performed using the Wilcoxon signed-rank test [56].

## Supporting information

S1 File Supplementary figure and table. S1 Fig illustrates the distinction between peripheral and non-peripheral inner nodes in the inner area municipality of Alberona. Table S1 presents the Pearson correlation values between *D*(*R*) and edge betweenness *b*(*R*) for all bridges in the Foggia province, evaluated across varying radius values *R*.
(PDF)
