# Supplementary material for: Data-driven assessment of Apulian road network resilience: Bridge unavailability and inner municipality isolation impact
Source: PLoS One. 2025 Oct 10;20(10):e0333308. doi: 10.1371/journal.pone.0333308 (PMC12513624; doi:10.1371/journal.pone.0333308)
Supplement: S1 File — S1 Fig illustrates the distinction between peripheral and non-peripheral inner nodes in the inner area municipality of Alberona. Table S1 presents the Pearson correlation values between D(R) and edge betweenness b(R) for all bridges in the Foggia province, evaluated across varying radius values R. (PDF) [file pone.0333308.s001.pdf]

S1 APPENDIX

Data-driven assessment of Apulian road network resilience: bridge unavailability and inner municipality isolation impact

Niloofar Kheirkhahan[1◑], Loredana Bellantuono[2,3◑], Nicola Amoroso[3,4], Roberto Cilli[1,3], Lorenzo De Biase[5], Valentina Lucaferri[6], Alfonso Monaco[1,3], Chiara Ormando[6], Ester Pantaleo[1,3*], Domenico Pomarico[1,3], Sabina Tangaro[3,7], Alberto Tofani[6‡], Roberto Bellotti[1,3‡]

1 Dipartimento Interateneo di Fisica “M. Merlin”, Università degli Studi di Bari Aldo Moro, Bari, Italy
2 Dipartimento di Biomedicina Traslazionale e Neuroscienze (DiBraiN), Università degli Studi di Bari Aldo Moro, Bari, Italy
3 Istituto Nazionale di Fisica Nucleare (INFN), Sezione di Bari, Università degli Studi di Bari Aldo Moro, Bari, Italy
4 Dipartimento di Farmacia - Scienze del Farmaco, Università degli Studi di Bari Aldo Moro, Bari, Italy
5 ENEA, Italian National Agency for New Technologies, Energy and Sustainable Economic Development, Bologna Research Center, Bologna, Italy
6 ENEA, Italian National Agency for New Technologies, Energy and Sustainable Economic Development, Casaccia Research Center, Rome, Italy
7 Dipartimento di Scienze del Suolo, della Pianta e degli Alimenti, Università degli Studi di Bari Aldo Moro, Bari, Italy

◑These authors contributed equally to this work. ‡These authors also contributed equally to this work. *ester.pantaleo@uniba.it

Abstract

This Appendix contains one table and one figure supporting the findings presented in the main text

Peripheral nodes

Figure S1 displays the municipality of Alberona, with its administrative boundary (black border) derived from ISTAT shapefiles [1]. The map highlights inner nodes within these borders, distinguishing them as peripheral or non-peripheral. Peripheral nodes are defined as those connected to at least one node outside the municipal boundary.

Comparing the relative efficiency drop with betweenness centrality

Table S1 reports the Pearson correlation between the relative efficiency drop and edge betweenness of the 369 bridges investigated in this study, for the considered values of the buffer radius R; for each correlation, the p-value is also provided.

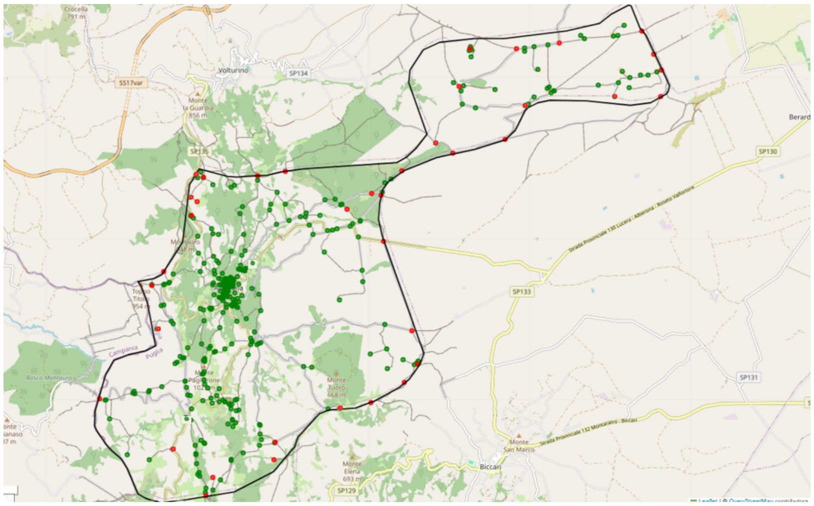

Figure S1. Inner nodes within Alberona municipality are classified as non-peripheral (green dots) or peripheral (red dots). Peripheral nodes are specifically those connected to at least one node outside the municipal boundary (black line). The municipal boundary shapefile is sourced from ISTAT [1], and the map is generated using the open-source Folium library [2].

Table S1. Pearson correlation coefficient (r) between the relative efficiency drop and edge betweenness of the 369 bridges, and corresponding p-value for different values of the buffer radius R.

**R (km)**	**Pearson correlation r**	**p-value**
5	-0.263	10^{-4}
10	-0.302	10^{-4}
15	-0.082	1.2×10^{-1}
20	-0.323	10^{-4}
25	-0.304	10^{-4}
30	-0.444	10^{-4}
35	-0.232	10^{-4}
40	-0.367	10^{-4}
45	-0.115	3.3×10^{-2}
50	-0.090	9.6×10^{-2}
60	-0.230	10^{-4}

References

1. ISTAT. Matrici del Pendolarismo; 2011. Accessed: 2024-09-23. https://www.istat.it/non-categorizzato/matrici-del-pendolarismo/.
2. Kolesnikov A. Folium v0.15.1; 2023. [cited 2025 Feb 5]. Available from: https://python-visualization.github.io/folium/.
